# CTA and DSA based computational fluid dynamics models for morphological and hemodynamic assessment of intracranial atherosclerotic stenosis

**DOI:** 10.3389/fneur.2025.1686189

**Published:** 2025-10-08

**Authors:** Rui Yang, Xulong Yin, Gaohui Li, Jianping Xiang, Qi Fang, Hui Wang, Bo Li

**Affiliations:** ^1^Department of Neurology, The First Affiliated Hospital of Soochow University, Suzhou, Jiangsu, China; ^2^Institute of Stroke Research, Soochow University, Suzhou, China; ^3^ArteryFlow Technology Co., Ltd., Hangzhou, China; ^4^Department of Interventional, The First Affiliated Hospital of Soochow University, Suzhou, Jiangsu, China

**Keywords:** intracranial atherosclerotic stenosis, computational fluid dynamics, hemodynamics, computed tomography angiography, digital subtraction angiograph

## Abstract

**Background:**

Intracranial atherosclerotic stenosis (ICAS) is a primary cause of ischemic stroke. Accurate assessment of anatomical and hemodynamic characteristics is crucial for treatment planning, yet current clinical evaluation primarily relies on luminal stenosis.

**Objective:**

This study aims to compare computational fluid dynamics (CFD) models based on digital subtraction angiography (DSA), computed tomography angiography (CTA) and CTA model incorporating DSA hemodynamic information (CMD) integrating DSA flow data with CTA morphological structure, evaluating their differences and consistency in ICAS assessment.

**Methods:**

40 ICAS patients who underwent CTA and DSA were retrospectively included. Patient-specific CFD simulations were performed using standardized boundary conditions to assess morphological data and hemodynamic parameters, including pressure ratio, wall shear stress ratio, and high shear stress areas. Statistical analyses included paired comparisons, intraclass correlation coefficients (ICC), and Bland–Altman analysis.

**Results:**

CTA-based models demonstrated excellent consistency with DSA in anatomical measurements (ICC > 0.90). The CMD approach enhanced consistency in functional metrics, with CMD-derived PR and WSSR highly concordant with DSA results. When using CTA alone, WSSR was slightly underestimated, particularly in middle artery lesions. Subgroup analysis indicated that lesion location significantly influences flow and shear stress patterns.

**Conclusion:**

CTA-based CFD modeling serves as a reliable non-invasive alternative to DSA for morphological ICAS assessment. The CMD method further improves the accuracy of functional evaluation by integrating flow data. These findings support the integration of anatomical imaging with hemodynamic modeling to enhance the clinical potential for stroke risk stratification.

## 1 Introduction

Ischemic stroke remains one of the leading causes of death and long-term disability among adults worldwide, imposing a substantial medical and economic burden on healthcare systems and society as a whole (1, 2). Intracranial atherosclerotic stenosis (ICAS) is one of the most common causes of ischemic stroke or transient ischemic attack (TIA) worldwide (3). Despite advances in medical management, patients with severe arterial stenosis (≥70%) continue to face a significantly elevated risk of stroke recurrence, particularly in the territory supplied by the stenotic vessel (4, 5). As a result, the degree of vascular stenosis has become a key determinant in clinical decision-making, particularly in guiding the need for endovascular interventions.

However, mounting evidence suggests that luminal stenosis alone may not be sufficient to accurately predict the risk of stroke recurrence (5, 6). Stroke pathogenesis is increasingly recognized as a multifactorial process that involves not only the severity of luminal narrowing but also other lesion-specific characteristics, including plaque composition and vulnerability, collateral circulation status, hemodynamic alterations, and downstream perfusion deficits (7–11). These additional factors offer the potential for more comprehensive and precise risk stratification.

In this context, the concept of functional assessment, originally developed for coronary artery disease using fractional flow reserve (FFR) (12–14) has been adapted to cerebrovascular disease through the development of fractional flow (FF) assessments for ICAS (15, 16). Concurrently, the application of computational fluid dynamics (CFD) in ICAS has emerged as a promising tool for noninvasive (17), patient-specific hemodynamic evaluation based on anatomical imaging modalities such as digital subtraction angiography (DSA) (18, 19), computed tomography angiography (CTA) (9, 20–22) and magnetic resonance angiography (MRA) (23–25).

Although many CFD studies have evaluated hemodynamic features in atherosclerotic lesions, most are limited to a single imaging modality, restricting cross-modal comparisons. As DSA and CTA are both widely used in clinical practice, a systematic comparison of CFD-derived morphological and hemodynamic parameters is necessary to understand their respective strengths. This study aimed to compare patient-specific CFD models based on DSA and CTA in ICAS, focusing on key parameters such as pressure gradient, wall shear stress, and flow patterns, to evaluate the consistency and reliability of functional assessments across modalities.

## 2 Materials and methods

### 2.1 Subjects and clinical data

**Figure 1:**
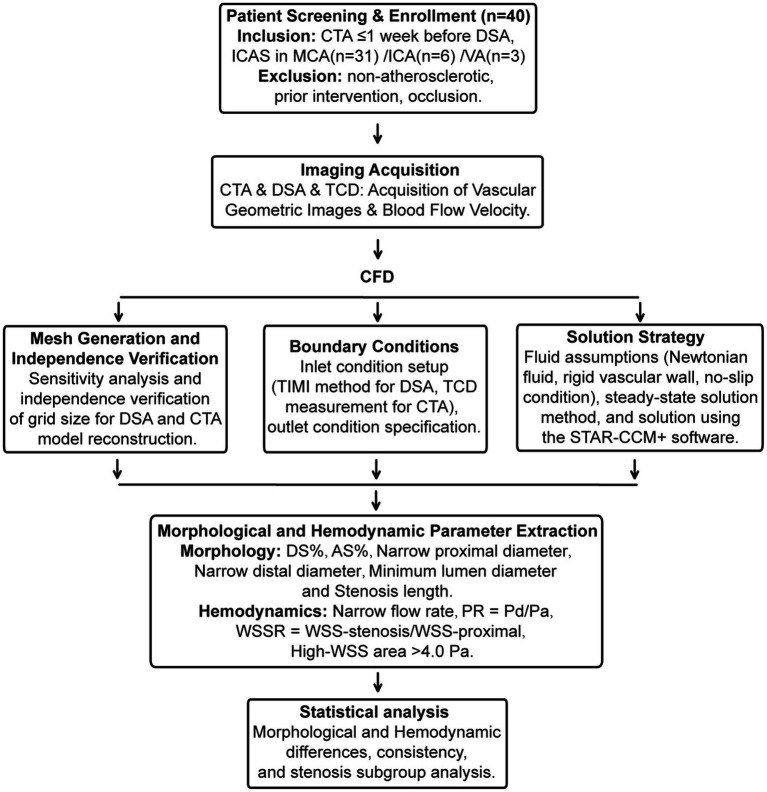
Flowchart of the present study. Flowchart of the present study. Involving the screening and enrollment of 40 patients, covering aspects such as image acquisition, CFD analysis, boundary condition setting, solution strategies, mesh generation, and extraction of morphological and hemodynamic parameters. Statistical analysis is subsequently conducted to explore morphological and hemodynamic differences, consistency, and subgroup analysis of stenosis.

A systematic review was conducted on the clinical data of 40 patients with ischemic stroke due to intracranial atherosclerotic stenosis, admitted to the First Affiliated Hospital of Soochow University between June 2021 and September 2022. The inclusion criteria were: (1) Computed tomography angiography (CTA) was performed within 1 week prior to Digital subtraction angiography (DSA); (2) Cerebral infarction caused by luminal stenosis of intracranial arteries, including the intracranial carotid arteries (ICA), the middle cerebral arteries (MCA), and the intracranial segments of the vertebrobasilar arteries (VA). Exclusion criteria included: (1) Ischemic stroke caused by factors other than atherosclerosis, such as Moyamoya disease, vasculitis, dissection, tumors, or cardioembolism; (2) Patients who had previously undergone interventional and/or surgical treatment of intracranial and extracranial arteries; (3) Cases of complete arterial occlusion (Figure 1). This study has received approval from the local Institutional Review Board (IRB) of the participating center. The data have been anonymized, and the requirement for informed consent has been waived.

### 2.2 Computed tomography angiography

The CT scanner selected is the Revolution 256 spiral CT machine from GE Company in the United States. CTA examination was conducted by injecting 40 mL of iodinated contrast agent and 50 mL of saline, both injected at a rate of 5 mL/s. Arterial and venous phase images were obtained with scanning parameters of 0.625 mm layer thickness, 512 × 512 matrix, and a scan range from the aortic arch to the top of the skull. The resolution of CTA is approximately 0.488 mm for each direction.

### 2.3 Digital subtraction angiography operation

We used Siemens Artis Zeego or Toshiba INFX-8000 V DSA machine as interventional imaging equipment. The right femoral artery was punctured using Seldinger modified technique, and a 4F H1 arterial sheath was inserted. Then, 2,000 U heparin was injected intravenously. A 5F pigtail catheter and a single curved catheter were used to perform angiography of the aortic arch, bilateral carotid arteries, and bilateral vertebral arteries, and contrast agent (Ioversol 320 mg/mL) was injected. The angiography parameters were as follows: injection rate 4-5 mL/s, injection dose 6-8 mL, shooting speed 6 frames/s. The average resolution of CTA is about 0.37 mm (0.35 mm ~ 0.39 mm).

### 2.4 Model reconstruction

The DICOM files of DSA and CTA of head vessels analyzed in this paper are all from the Neusoft Imaging Workstation of the First Affiliated Hospital of Soochow University. First, the image data in DICOM format was imported into MIMICS, Version 20.0, for image segmentation and reconstruction (26). Removing side branches (radius < 50% of parent vessel) has negligible impact on PR measurement accuracy (27), but unknown effect on other parameters, so retained stenotic peripheral branches where possible.

### 2.5 Hemodynamic simulation of CFD

#### 2.5.1 Mesh generation and Independence verification

Polyhedral volume meshs with prism layers near the wall were generated for each model. To ensure the accuracy of the simulation results was independent of mesh density, a mesh independence study was conducted on a representative model with 62% MCA diameter stenosis. The translesional pressure ratio (PR) was monitored as the key metric. The solution was deemed mesh-independent when the PR value varied by less than 2% upon further refinement. Based on this study, maximum cell size of 0.15 mm was adopted for all simulations, resulting in approximately 1 to 2 million cells per model.

#### 2.5.2 Boundary conditions

For models derived from DSA images, the patient-specific time-averaged flow velocity at the inlet boundary was determined using the TIMI frame count method (27) and converted to a flow rate. A constant velocity-inlet boundary condition was prescribed at the model inlet. For models derived from CTA images, the patient-specific time-averaged flow velocity at the inlet boundary was obtained from Transcranial Doppler (TCD) ultrasound measurements of the relevant artery (28). TCD examinations were conducted within 48 h of patient admission by two certified neurosonographers, utilizing a transcranial Doppler device (EMS-9 PB; Shenzhen Delica Medical Equipment Co., Ltd., Shenzhen, China). The mean flow velocities of the middle cerebral artery, intracranial segments of the internal carotid artery, and the proximal portion of the vertebrobasilar artery were measured, with three independent measurements averaged to minimize operational error. The resulting patient-specific mean flow velocity values were employed as inlet boundary conditions for the model. All outlets were modeled as split outlet boundary conditions with flow rates proportional to the cube of their diameters. The CTA model incorporating DSA hemodynamic information (CMD) used the same geometry and outlet conditions as the CTA group, but with the inlet flow rate replaced by the DSA-derived TIMI frame count value.

#### 2.5.3 Solution strategy

Patient-specific models derived from DSA and CTA were simulated using the finite volume method (FVM) in STAR-CCM + v.20.02 (Siemens Digital Industries Software, Plano, TX, USA). Blood was modeled as an incompressible Newtonian fluid (density = 1,060 kg/m^3^, viscosity = 0.0035 Pa·s) with rigid, no-slip vessel walls (9, 20, 21). Steady-state simulations under mean-flow conditions were performed to assess translesional pressure drop, neglecting pulsatility effects. The SIMPLE algorithm coupled pressure and velocity, with second-order spatial discretization. Convergence was achieved when normalized residuals for continuity and momentum fell below 10^−5^. The blood flow simulation method similar to DSA has recently been validated by comparing the simulated values with pressure wire measurements in the literature (19).

#### 2.5.4 Definition of relevant parameters

The comparative evaluation of DSA and CTA for stenotic vessels focuses on two aspects. Firstly, the morphological evaluation mainly includes six anatomical parameters, percentage of diameter stenosis (DS%) (29), percentage of area stenosis (AS%), narrow proximal diameter, narrow distal diameter, minimum lumen diameter (30) and stenosis length. Secondly, the hemodynamic evaluation includes four parameters, Narrow flow rate, translesional pressure ratio (PR): defined as the ratio of mean pressure distal to the stenosis to that proximal to the stenosis (PR = P_distal / P_proximal) (9, 22), wall shear stress ratio (WSSR): defined as the ratio of mean wall shear stress within the stenotic throat to the mean wall shear stress in the proximal reference segment (WSSR = WSS_stenosis / WSS_proximal) (9, 23) and the area of high wall shear stress (High-WSS area): defined as the luminal surface area exposed to wall shear stress values greater than 3.0 Pa, a threshold widely used to indicate abnormally elevated shear stress in intracranial and carotid arteries (20).

### 2.6 Statistical analysis

DSA is recognized as the gold standard for detecting vascular stenosis (30). Consequently, in this study, the DSA-based model was established as the “standard model,” and the CTA-based model along with the CMD were compared against the “standard model DM.” A comprehensive statistical analysis was conducted to evaluate the morphological and hemodynamic consistency among DSA, CTA, and CMD. For continuous variables, the Shapiro–Wilk test was employed to assess normality. Depending on the data distribution, paired t-tests or Wilcoxon signed-rank tests were used for comparisons between two groups, while the Kruskal-Wallis test was applied for comparisons among three groups with non-normally distributed data. To assess the agreement between imaging modalities, Bland–Altman plots were utilized to evaluate consistency, and the absolute agreement ICC were calculated using a two-way random effects model. All statistical tests were two-sided, with a *p*-value < 0.05 considered statistically significant.

## 3 Results

### 3.1 Baseline characteristics

A total of 40 patients with intracranial arterial stenosis were included. The most frequently affected vascular territory was the MCA, accounting for 77.5% (31/40) of cases, followed by the ICA (15%) and the vertebrobasilar artery VA (7.5%). The mean fasting blood glucose level was 6.45 ± 1.93 mmol/L, with a mean triglyceride level of 1.42 ± 0.76 mmol/L and a mean HbA1c of 6.38 ± 1.07%. The levels of high-density lipoprotein (HDL) and low-density lipoprotein cholesterol (LDL-C) were 1.12 ± 0.31 mmol/L and 2.40 ± 0.77 mmol/L, respectively. Regarding neurological status, the median modified Rankin Scale (mRS) score prior to admission was 1 (interquartile range [IQR]: 0–2), and the median National Institutes of Health Stroke Scale (NIHSS) score at admission was 0 (IQR: 0–2). In terms of medical history, 29 patients (72.5%) had a history of hypertension, 16 (40.0%) had dyslipidemia, and 9 (22.5%) had diabetes. Six patients (15.0%) were current or former smokers, 5 (12.5%) had a history of ischemic heart disease, and 23 patients (57.5%) had a prior history of stroke or TIA.

### 3.2 Morphology data analysis of DSA and CTA

#### 3.2.1 Analysis of morphological assessment differences

To assess the concordance between DSA and CTA in morphological measurements (Table 1), we conducted a paired statistical comparison of key vascular indices, including diameter stenosis, area stenosis, proximal and distal reference diameters of narrow, minimal lumen diameter, and stenosis length. Across all parameters, the measurements from DSA and CTA exhibited no statistically significant differences. Specifically, the median diameter stenosis rate in the DSA group was 59.95% [IQR: 49.35–76.50], whereas in the CTA group, it was 58.95% ± 19.84 (*p* = 0.618). For area stenosis, the DSA yielded 82.98% [71.52–94.00], compared to 83.56% [71.97–92.94] from CTA (*p* = 0.510). Similarly, no significant discrepancies were observed in proximal diameter (DSA: 2.29 [1.90–2.74] mm; CTA: 2.25 [1.86–2.85] mm; *p* = 0.154) or distal diameter (DSA: 2.23 ± 0.72 mm; CTA: 2.26 ± 0.73 mm; *p* = 0.068). Likewise, minimal lumen diameter (DSA: 1.02 ± 0.52 mm; CTA: 1.05 ± 0.55 mm; *p* = 0.509) and stenosis length (DSA: 4.28 ± 2.18 mm; CTA: 4.43 ± 2.27 mm; *p* = 0.164) did not demonstrate significant differences. These findings indicate a high level of agreement between DSA and CTA in evaluating the morphological aspects of intracranial stenosis, suggesting that CTA can serve as a reliable non-invasive alternative for morphological assessment in clinical practice.

**Table 1:** Morphological differences analysis between DSA and CTA.

Indicator	DSA	CTA	*P*-value
Diameter Stenosis (%)	59.95 [49.35, 76.50]	57.77 [49.34, 74.18]	0.618
Area Stenosis (%)	82.98 [71.52, 94.00]	83.56 [71.97, 92.94]	0.510
Narrow Proximal Diameter (mm)	2.29 [1.90, 2.74]	2.25 [1.86, 2.85]	0.154
Narrow Distal Diameter (mm)	2.23 (0.72)	2.26 (0.73)	0.068
Minimal Lumen Diameter (mm)	1.02 (0.52)	1.05 (0.55)	0.509
Stenosis Length (mm)	4.28 (2.18)	4.43 (2.27)	0.164

CTA, CT angiography; DSA, digital subtraction angiography.

#### 3.2.2 Morphological assessment consistency analysis

**Figure 2:**
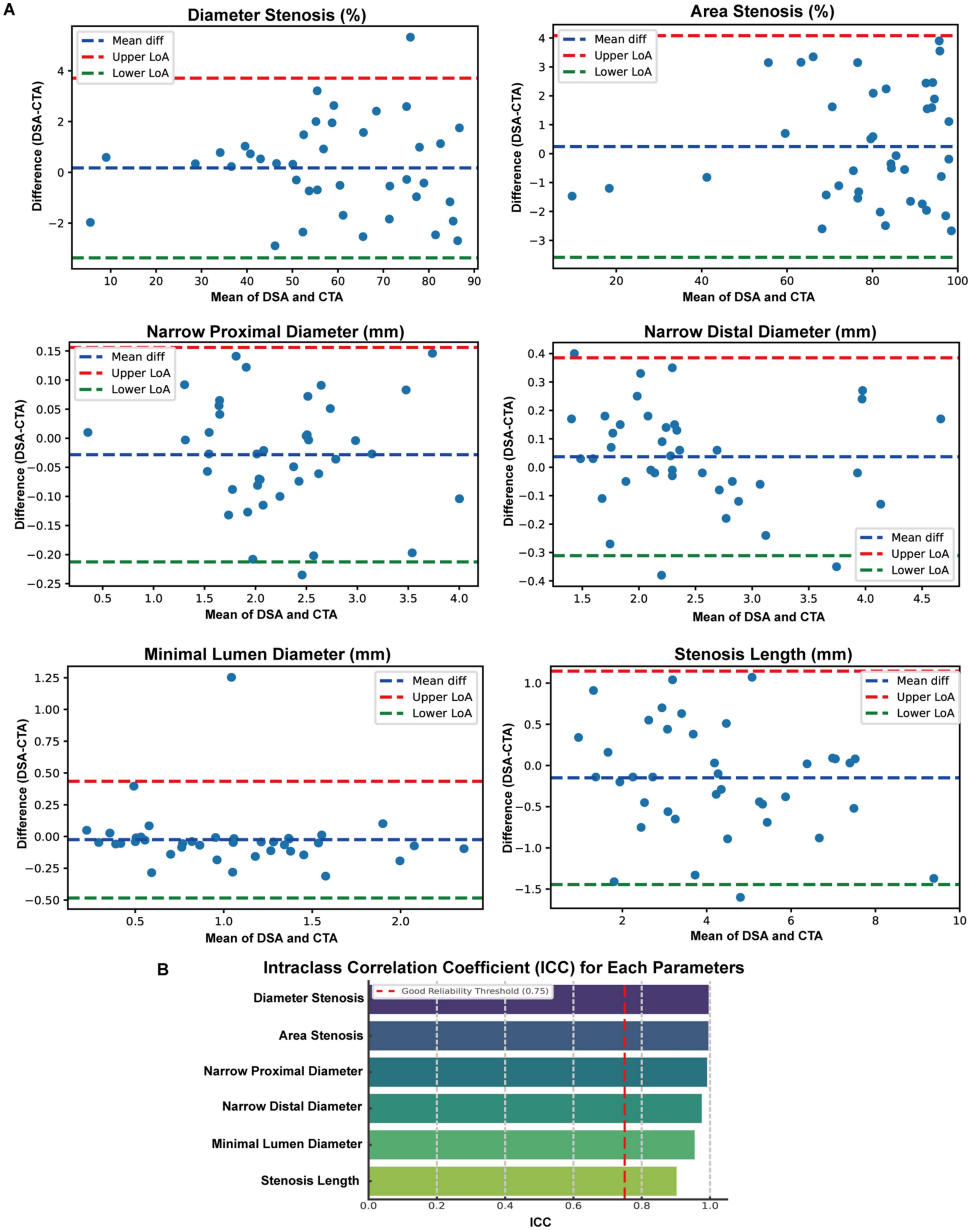
Consistency Analysis of Morphological Parameters between DSA and CTA. **(A)** Bland-Altman plots were employed to compare six paired anatomical measurements obtained from digital subtraction angiography (DSA) and computed tomography angiography (CTA), encompassing percent diameter stenosis, percent area stenosis, proximal diameter, distal diameter, minimal lumen diameter, and stenosis length. The blue dashed lines represent the mean differences, while the red and green dashed lines denote the Upper and Lower 95% limits of agreement (LoA), respectively. **(B)** Intraclass correlation coefficient (ICC) analysis results for the same six parameters.

To evaluate the consistency between DSA and CTA in assessing vascular morphology, we performed Bland–Altman and ICC analyses across six paired anatomical parameters. Bland–Altman plots demonstrated that for all parameters (Figure 2A), the mean differences between DSA and CTA measurements were small, with most values falling within the 95% limits of agreement (LoA), indicating no significant systematic bias. Specifically, the mean differences (DSA – CTA) were: Percentage of Diameter Stenosis: 0.17, with LoA ranging from −10.71 to 11.05; Percentage of Area Stenosis: 0.25, with LoA from −9.57 to 10.06; Proximal Diameter: 0.04 mm, LoA from −0.62 to 0.71 mm; Distal Diameter: −0.03 mm, LoA from −0.60 to 0.54 mm; Minimum Lumen Diameter: −0.03 mm, LoA from −0.52 to 0.46 mm; Stenosis Length: −0.15 mm, LoA from −2.67 to 2.36 mm. These results suggest that individual-level variation between DSA and CTA was acceptable and showed no systematic measurement shift. Furthermore, intraclass correlation analysis using a two-way random-effects model revealed excellent agreement between DSA and CTA (Figure 2B): Percentage of Diameter Stenosis: ICC = 0.996; Percentage of Area Stenosis: ICC = 0.995; Proximal Diameter: ICC = 0.976; Distal Diameter: ICC = 0.991; Minimum Lumen Diameter: ICC = 0.902; Stenosis Length: ICC = 0.985; All ICC values exceeded 0.90, indicating excellent consistency between DSA and CTA across all morphological measurements. These findings confirm that CTA-based vascular modeling can serve as a reliable alternative to DSA for quantifying anatomical features relevant to intracranial arterial stenosis.

#### 3.2.3 Morphological subgroup analysis

Subgroup analysis was conducted to compare morphological characteristics of intracranial atherosclerotic lesions located in the MCA (*n* = 31), ICA (*n* = 6), and VA (*n* = 3), using both DSA and CTA (Table 2). For the percentage of diameter stenosis, both DSA and CTA showed no statistically significant differences across vascular territories (DSA: MCA 60.30 [47.71–77.62], ICA 56.27 ± 14.81, VA 65.71 ± 7.51; *p* = 0.655; CTA: MCA 58.97 ± 21.47, ICA 56.10 ± 15.51, VA 64.45 ± 9.52; *p* = 0.845). Similarly, the percentage of area stenosis was not significantly different between regions in either modality (DSA: MCA 84.20 [69.92–95.03], ICA 79.07 ± 10.65, VA 86.44 ± 5.39; *p* = 0.725; CTA: MCA 84.29 [69.82–93.34], ICA 77.74 ± 10.25, VA 87.37 ± 6.69; *p* = 0.607). In contrast, proximal vessel diameter showed significant differences across all territories for both imaging methods (DSA: MCA 2.19 ± 0.48 mm, ICA 3.26 ± 0.87 mm, VA 3.95 ± 0.89 mm; *p* < 0.01; CTA: MCA 2.15 ± 0.57 mm, ICA 3.26 ± 0.85 mm, VA 3.89 ± 0.67 mm; p < 0.01). A similar pattern was observed for the distal diameter, which was significantly larger in the ICA and VA compared to the MCA (DSA: MCA 2.00 ± 0.55 mm, ICA 2.78 ± 0.74 mm, VA 3.46 ± 0.49 mm; *p* < 0.01; CTA: MCA 2.04 ± 0.57 mm, ICA 2.73 ± 0.68 mm, VA 3.56 ± 0.54 mm; p < 0.01). Although the minimum lumen diameter tended to be larger in ICA and VA lesions, the differences did not reach statistical significance in either modality (DSA: MCA 0.93 ± 0.48 mm, ICA 1.35 ± 0.59 mm, VA 1.33 ± 0.62 mm; *p* = 0.105; CTA: MCA 0.94 ± 0.51 mm, ICA 1.39 ± 0.60 mm, VA 1.46 ± 0.57 mm; *p* = 0.071). Overall, both DSA and CTA consistently revealed that while stenosis severity (percent narrowing) remained comparable among vascular segments, vessel caliber—particularly proximal and distal diameters—differed significantly among MCA, ICA, and VA lesions. This suggests that anatomic location should be considered when evaluating lesion morphology or planning interventional strategies.

**Table 2:** Morphological subgroup analysis of DSA and CTA.

Morphological parameters	Source	MCA (*n* = 31)	ICA (*n* = 6)	VA (*n* = 3)	*P*-value
Diameter Stenosis (%)	DSA	60.30 [47.71–77.62]	51.73 [47.56, 58.45]	69.67 [63.36, 70.03]	0.655
	CTA	58.97 ± 21.47	56.10 ± 15.51	64.45 ± 9.52	0.845
Area Stenosis (%)	DSA	84.20 [69.92–95.03]	76.67 [72.48, 82.65]	88.10 [84.25, 89.45]	0.725
	CTA	84.29 [69.82–93.34]	75.39 [73.24, 82.34]	89.75 [84.78, 91.15]	0.607
Narrow Proximal Diameter (mm)	DSA	2.19 ± 0.48	3.26 ± 0.87	3.95 ± 0.89	<0.01
	CTA	2.15 ± 0.57	3.26 ± 0.85	3.89 ± 0.67	<0.01
Narrow Distal Diameter (mm)	DSA	2.00 ± 0.55	2.78 ± 0.74	3.46 ± 0.49	<0.01
	CTA	2.04 ± 0.57	2.73 ± 0.68	3.56 ± 0.54	<0.01
Minimal Lumen Diameter (mm)	DSA	0.93 ± 0.48	1.35 ± 0.59	1.33 ± 0.62	0.105
	CTA	0.94 ± 0.51	1.39 ± 0.60	1.46 ± 0.57	0.071
Stenosis Length (mm)	DSA	4.09 ± 2.09	4.80 ± 2.81	5.19 ± 2.18	0.594
	CTA	4.17 [2.63, 5.83]	3.71 [2.83–4.49]	5.78 [5.08, 6.58]	0.396

CTA, CT angiography; DSA, digital subtraction angiography; MCA, middle cerebral artery; ICA, internal carotid artery; VA, vertebral-basilar artery.

### 3.3 Hemodynamic comparison of DSA and CTA

#### 3.3.1 Hemodynamic assessment differential analysis

**Figure 3:**
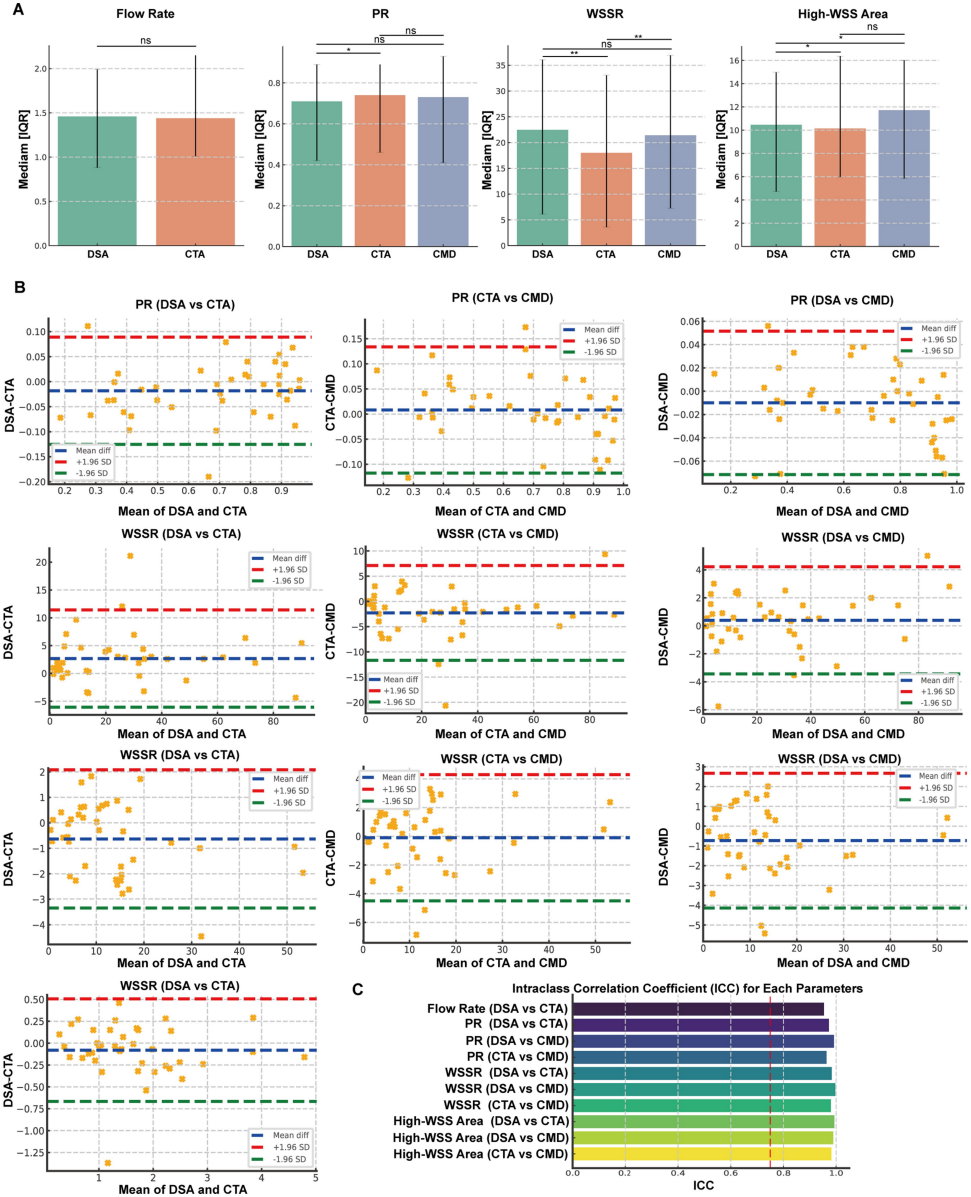
Comparative Analysis and Consistency Evaluation of Hemodynamic Parameters Derived from DSA, CTA, and CMD Models. **(A)** Dual Comparisons of Flow, Pressure Ratio (PR), Wall Shear Stress Ratio (WSSR), and High WSS Areas Among Digital Subtraction Angiography (DSA), Computed Tomography Angiography (CTA), and CMD. Box plots illustrate the median and interquartile range. **(B)** Bland-Altman Plots and Intraclass Correlation Coefficient (ICC) Analysis for Flow, PR, and WSSR Between Each Imaging Mode Pair (DSA vs. CTA, DSA vs. CMD, CTA vs. CMD). The blue dashed line represents the mean difference, while the red and green dashed lines indicate the Upper and Lower 95% Limits of Agreement (LoA), respectively. **(C)** ICC Analysis Results for Hemodynamic Parameters. Significance levels are marked as *p < 0.05, **p < 0.01, ***p < 0.001, ns: not significant.

To comprehensively compare hemodynamic parameters obtained from DSA, CTA, and CMD, pairwise comparisons were performed for flow rate, pressure ratio (PR), wall shear stress ratio (WSSR), and High WSS area (Figure 3A). For flow rate, the median (IQR) values were 1.46 (0.88–1.99) ml/s for DSA and 1.44 (1.01–2.15) ml/s for CTA, with no significant difference (*p* = 0.116). For PR, the median values were 0.71 (0.42–0.89) for DSA, 0.74 (0.46–0.89) for CTA, and 0.73 (0.41–0.93) for CMD. Significant differences were observed between DSA and CTA (*p* = 0.041), whereas DSA vs. CMD (*p* = 0.068) and CTA vs. CMD (*p* = 0.452) showed no significant difference. For WSSR, the median values were 22.49 (6.11–36.10) for DSA, 18.02 (3.58–33.07) for CTA, and 21.43 (7.25–36.94) for CMD. Significant differences were noted between DSA and CTA (*p* < 0.01) and between CTA and CMD (*p* = 0.002), while no significant difference was found between DSA and CMD (*p* = 0.109). This suggests CMD-derived WSSR is closer to DSA estimates than CTA-derived values. For High WSS area, the values were 10.46 (4.72–14.98) for DSA, 10.15 (5.97–16.37) for CTA, and 11.72 (5.85–16.02) for CMD. Differences were statistically significant between DSA and CTA (*p* = 0.014) and between DSA and CMD (*p* = 0.019), but not between CTA and CMD (*p* = 0.735). Overall, hemodynamic measurements from DSA, CTA, and CMD demonstrated good consistency, although WSSR and high WSS area exhibited method-dependent variability.

#### 3.3.2 Hemodynamic assessment consistency analysis

To evaluate the measurement consistency of hemodynamic parameters obtained by DSA, CTA, and CMD, we conducted intra-group correlation coefficient (ICC) analysis and Bland–Altman consistency assessment for four key indicators PR, WSSR, and high WSS area (Figure 3B). In terms of traffic, the results show that DSA and CTA have excellent consistency, with an ICC value of 0.954. The Bland–Altman plot shows the mean difference is −0.080 mL/s and the 95% consistency limit (LoA) range is −0.667 to 0.506 mL/s. For PR, all three methods showed strong commonality. The ICC values are 0.973 (DSA vs. CTA), 0.992 (DSA vs. CMD), and 0.964 (CTA vs. CMD), respectively. Bland–Altman analysis showed that the mean difference was very small, which was −0.018, −0.010 and +0.008, respectively, and the consistency boundary interval was narrow (such as the LoA of DSA and CMD was −0.072 to 0.052), indicating that the methods were highly consistent and had small deviations. In terms of WSSR, the ICC values also show excellent reliability: 0.983 (DSA vs. CTA), 0.989 (DSA vs. CMD), and 0.988 (CTA vs. CMD). The corresponding mean differences were 2.67, −0.77, and −3.45, with the consistency boundary ranging from about −6.08 to 11.41 for DSA and CTA to −11.99 to 5.09 for CTA and CMD. These results show that the WSSR estimates provided by CMD are more consistent with DSA than CTA. The intraclass correlation coefficient (ICC) demonstrated excellent agreement between measurement methods across all parameters, with values ranging from 0.902 to 0.996. The highest consistency was observed for diameter stenosis (ICC = 0.996) and area stenosis (ICC = 0.995), while minimum lumen diameter showed the lowest, yet still excellent, agreement (ICC = 0.902). All ICC values exceeded the 0.75 threshold for good reliability, and most surpassed the 0.90 threshold for excellent reliability (Figure 3C).

#### 3.3.3 Hemodynamic subgroup analysis

**Figure 4:**
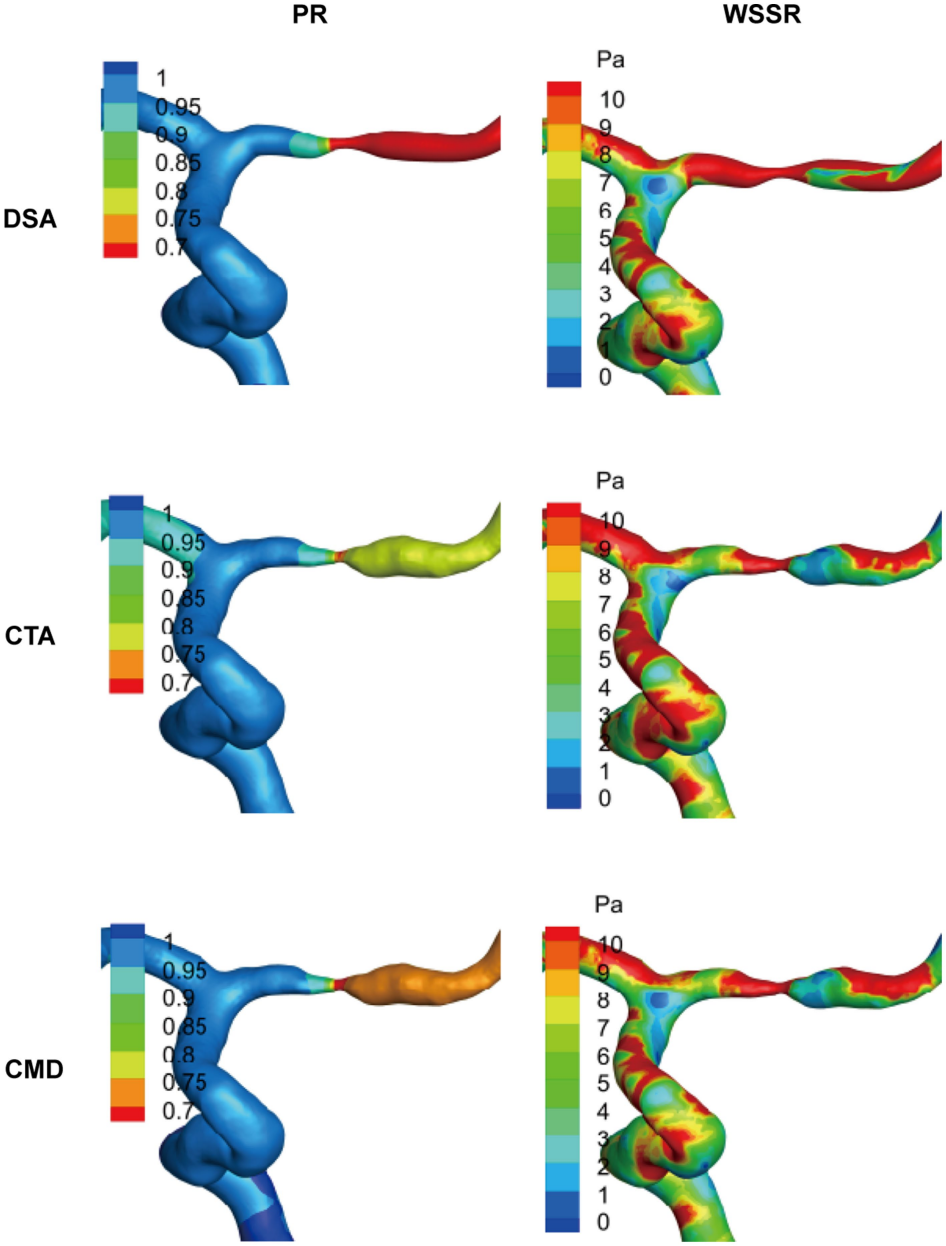
Representative visualization of hemodynamic analysis in cases of MCA stenosis. Based on the results of patient-specific computational fluid dynamics (CFD) analysis of typical middle cerebral artery (MCA) lesions, the spatial distributions of pressure ratio (PR) and wall shear stress ratio (WSSR) are presented. These data are derived from digital subtraction angiography (DSA), computed tomography angiography (CTA), and their combined model (CMD). The color maps along the vessel surface indicate the intensity of each parameter, highlighting the specific differences in hemodynamic assessment among the various imaging modalities.

To investigate the impact of the lesion site on hemodynamic characteristics, the study continued to categorize the stenosis based on its anatomical location into MCA (*n* = 31), VA (*n* = 3), and ICA (*n* = 6). Comparative analyses were conducted across these anatomical subgroups (Table 3). In terms of flow rate, both DSA and CTA data revealed significant differences among the three vascular regions (*p* values of 0.006 and 0.001, respectively), with the ICA lesion group exhibiting the highest mean flow rate (DSA: 2.86 ± 1.04 mL/s; CTA: 2.97 ± 1.09 mL/s), followed by the VA and MCA groups. Regarding PR, although CMD-derived PR tended to be lower in MCA lesions compared to VA and ICA, no statistically significant differences were observed among the three methods (DSA: *p* = 0.175; CTA: *p* = 0.205; CMD: *p* = 0.184). For WSSR, CTA identified significant intergroup differences (*p* = 0.040), with the VA lesion group showing the highest mean WSSR (36.06 ± 3.53), while the MCA and ICA groups exhibited more dispersed distributions. Although comparisons of WSSR based on DSA and CMD did not reach statistical significance (*p* > 0.05), they exhibited similar trends. Significant differences in high WSS regions were observed across anatomical locations in all three imaging modalities (DSA: *p* < 0.01; CTA: *p* < 0.01; CMD: *p* = 0.049). VA and ICA lesions consistently demonstrated larger high WSS areas (CTA: VA = 27.59 ± 8.03 mm^2^ vs. MCA = 9.59 ± 6.19 mm^2^), indicating more extensive local hemodynamic disturbances compared to MCA stenosis. In summary, these findings demonstrate that the stenosis location has a measurable influence on hemodynamic parameters, particularly flow magnitude and WSS distribution. Lesions in proximal arteries such as ICA and VA are often associated with higher flow rates and larger high WSS regions, which may reflect differences in vessel caliber, collateral circulation supply, and compensatory hemodynamic adjustments. As representatives, we present the hemodynamic analysis visualization of PR and WSSR using MCA in Figure 4.

**Table 3:** Hemodynamic subgroup analysis of DSA and CTA.

Hemodynamic parameters	Source	MCA (*n* = 31)	ICA (*n* = 6)	VA (*n* = 3)	*P* value
Flow rate	DSA	1.28 [0.78–1.65]	2.37 [2.20–3.44]	1.34 [1.08–1.86]	0.006
	CTA	1.41 ± 0.78	2.97 ± 1.09	1.69 ± 0.67	0.001
FFR	DSA	0.65 [0.37–0.86]	0.81 [0.71–0.87]	0.90 [0.82–0.94]	0.175
	CTA	0.69 [0.44–0.86]	0.84 [0.75–0.88]	0.90 [0.82–0.92]	0.205
	CMD	0.61 [0.40–0.88]	0.81 [0.73–0.87]	0.94 [0.84–0.97]	0.185
WSSR	DSA	23.20 [8.94–36.66]	5.60 [3.00–8.91]	32.04 [31.91–37.69]	0.155
	CTA	18.19 [5.50–31.84]	2.24 [2.16–2.62]	35.24 [33.72–37.99]	0.040
	CMD	22.59 [9.19–37.25]	7.62 [4.32–9.32]	35.57 [32.41–39.19]	0.227
High-WSS Area	DSA	9.30 ± 6.11	24.60 ± 21.45	25.02 ± 9.54	<0.01
	CTA	9.59 ± 6.30	26.06 ± 21.49	27.59 ± 9.83	<0.01
	CMD	8.51 [5.36–15.12]	14.00 [11.50–42.48]	31.30 [22.32–31.99]	0.049

PR, pressure ratio; WSSR, wall shear stress ratio; High-WSS area, the area of high wall shear stress.

## 4 Discussion

This study systematically compared patient-specific CFD models constructed based on DSA, CTA, and CMD that integrates DSA blood flow data with CTA geometric morphology, in the assessment of morphological and hemodynamic characteristics of ICAS. The results demonstrated high consistency among the modalities in both anatomical and functional parameters, further validating the potential of CTA-based CFD as a non-invasive alternative to DSA for clinical evaluation.

The patient cohort included in this study predominantly featured intracranial arterial lesions and commonly exhibited traditional vascular risk factors such as hypertension and dyslipidemia, indicating that our sample population is representative within the context of ICAS research. DSA, long regarded as the “gold standard” for clinical diagnosis of vascular stenosis, has faced obstacles in its widespread adoption due to its high cost, invasive nature, and an estimated 1–2% incidence of procedure-related morbidity (31). Among the current non-invasive imaging methods, CTA has gained widespread adoption and application due to its high discriminatory power in identifying severe stenosis and occlusions (32–35). At the morphological level, CTA and DSA exhibit highly concordant measurements of stenosis severity, reference diameters, and lesion lengths, with intraclass correlation coefficients (ICC > 0.90) demonstrating strong agreement. Additionally, the Bland–Altman plots indicate minimal measurement bias. These findings substantiate CTA as a reliable imaging modality for assessing the luminal morphology of ICAS, consistent with prior studies that highlight its high diagnostic accuracy for moderate to severe intracranial stenosis (9, 36, 37). However, subtle discrepancies have been observed in certain cases. CTA tends to slightly overestimate the distal reference diameter or the area of stenosis, a phenomenon potentially attributable to partial volume effects, calcification artifacts, or segmentation threshold variability, particularly in small-caliber vessels such as the MCA (35, 38, 39). Although these differences did not achieve statistical significance at the group level, they may influence therapeutic decision-making for critical lesions, thereby underscoring the critical importance of cautious interpretation of CTA measurements in clinical practice.

Compared to other hemodynamic assessment methodologies, the establishment of CFD necessitates the input of boundary blood flow information, which is often challenging to obtain under non-invasive conditions. The circulatory system within the human vascular network operates dynamically, influenced by a multitude of factors including instantaneous blood pressure, blood viscosity, collateral circulation, and vascular wall elasticity (40, 41). The assumptions regarding the boundary conditions and hemodynamic parameters within the CFD model significantly influence the accuracy of simulated blood flow. Wang et al. employed the TIMI frame count method, integrating DSA imaging with a three-dimensional model to compute the PR at the distal and proximal sites of intracranial stenosis, as well as the pressure wire measurements, achieving a strong correlation (r = 0.908, *p* < 0.001) (19). Liu et al. enhanced the consistency between CFD and pressure wire measurements by iteratively adjusting the inlet pressure boundary conditions after each CFD computation (22). However, in previous studies, CFD models based on CTA (9, 20, 21) or MRA (23) typically employed fixed flow rate information directly, which may introduce computational errors. Chen et al. integrated the stenotic area and blood flow velocity obtained from transcranial Doppler with MRA data and found that the WSSR exhibited a significant correlation with the degree of stenosis (25). This study utilized the TIMI method or neck vascular ultrasound to obtain specific flow rates at corresponding stenotic sites (42).

Previous studies have demonstrated that the morphological assessment of intracranial stenosis only partially reflects the physiological severity in patients with distal cerebral flow restriction (43, 44), whereas the PR quantifies the pressure gradient across the stenosis. In the present study, excellent concordance was observed in flow and PR measurements among DSA, CTA, and CFD simulations, with an intraclass correlation coefficient (ICC > 0.95). This finding substantiates the high reliability of CTA-based simulations when boundary conditions derived from DSA are incorporated. Previous studies have established that WSS, as a hemodynamic parameter, is associated with the degree of vascular stenosis and the pressure gradient. Elevated WSS (45, 46), on one hand, promotes the formation of atherosclerotic plaques, while on the other hand, it induces plaque rupture and thrombus formation. This study incorporated fixed quantitative WSSR and high WSS regions for comparative analysis. In terms of the WSSR, the results from CMD often exhibit closer alignment with DSA measurements compared to those obtained using CTA alone. Nonetheless, WSSR demonstrates the highest variability associated with imaging modalities, particularly in lesions of the MCA. This variability can be attributed to the complex geometry and flow dynamics near the proximal stenotic segment, especially around bifurcations, which complicates the definition of prestenotic WSS. As a ratio-based metric, WSSR is highly sensitive to minor variations in the denominator, thereby magnifying measurement errors. In contrast, high WSS regions, serving as a more stable and spatially integrated parameter, maintain consistency across different imaging modalities and vascular regions. Notably, previous studies have established a correlation between larger high WSS regions and plaque regression or vulnerability, reinforcing their significance as functional biomarkers of disease progression (24, 47).

Subgroup analysis demonstrates that the location of the lesion significantly influences both morphological and hemodynamic characteristics. Compared to MCA stenosis, lesions in the ICA and VA exhibit larger proximal and distal vessel diameters, higher blood flow rates, and more extensive regions of high WSS. These differences likely reflect the anatomical distinctions between proximal and distal cerebrovascular segments and the compensatory hemodynamic alterations that ensue (19, 21, 48). Interestingly, WSSR exhibited significant inter-regional variability exclusively in CTA-based models, suggesting that CTA-derived WSSR may be more susceptible to geometric distortions or segmentation variability. The feasibility of CMD as a hybrid approach warrants special mention. By integrating CTA’s high spatial resolution with the accurate flow dynamics derived from DSA, CMD offers a promising compromise between accessibility and precision. In this study, PR and WSSR derived from CMD demonstrated high concordance with DSA results, corroborating previous literature that integrating reliable flow data into non-invasive geometric models can significantly enhance the accuracy of CFD (21, 49).

Several limitations are inherent in this study: Initially, the small sample size necessitates the inclusion of a larger patient cohort to validate the accuracy of the findings, despite the preliminary explorations offering feasibility for subsequent in-depth investigations. Secondly, not all patients underwent all imaging examinations on the same day, implying that the luminal structure of the diseased vessels and the associated hemodynamic information may have varied temporally. Additionally, when employing measurements such as TIMI frame count or TCD to quantify patient-specific hemodynamic data, uncertainties arising from operator variability or imaging quality can introduce measurement errors. Lastly, akin to all previous CFD models, the simplified blood flow simulation model utilized in this study overlooks confounding factors such as inlet blood pulsatility and collateral circulation, which to some extent compromise the precision of vascular CFD simulation results. Future research should address these factors. It is crucial to carefully consider these limitations when interpreting the study outcomes.

## 5 Conclusion

This study demonstrates that the CTA-based CFD model exhibits a high degree of consistency with DSA in the anatomical and hemodynamic assessment of intracranial atherosclerotic stenosis. CTA model incorporating DSA hemodynamic information significantly enhances the accuracy of functional indices such as PR and WSSR. Although CTA alone may underestimate WSSR in the evaluation of middle cerebral artery lesions, it remains reliable in assessing the degree of stenosis and regions of high WSS. These findings support the use of CTA-based CFD technology as a feasible and non-invasive alternative to DSA, highlighting the potential of CMD in improving the precision of functional assessments.

## Data Availability

The raw data supporting the conclusions of this article will be made available by the authors on request. Requests to access these datasets should be directed to Xulong Yin, yxl15662190886@163.com.
